# A national training course for clinical trainers in family medicine

**DOI:** 10.4102/phcfm.v16i1.4341

**Published:** 2024-01-04

**Authors:** Hanneke Brits

**Affiliations:** 1 Department of Family Medicine, Faculty of Health Sciences, University of the Free State, Bloemfontein, South Africa

**Keywords:** clinical trainers, family medicine, workplace-based training, adult learning, face-to-face training

## Abstract

Quality vocational training is important to ensure that family physicians can strengthen the district health system. Competent clinical trainers and supervisors, with the necessary educational knowledge and skills, are required for this job. In 2014, a formal Train the Clinical Trainer (TCT) course was introduced in South Africa as a collaborative effort between the Royal College of General Practitioners (RCGP) and the South African Academy of Family Physicians (SAAFPs). To make the training relevant, the course was aligned with the National Unit Standards for Family Medicine, as these defined the learning outcomes for registrars. The aim of the TCT course was to equip workplace-based clinical trainers with an essential set of educational skills, which could be further developed through mentoring and support. Accredited trainers present the face-to-face course yearly to two family physicians from each of the nine departments of family medicine in South Africa. The TCT course is built around the educational principles of learner-centredness and andragogy. During these 5 days of training the roles and responsibilities of trainers and learners, the learning environment, alignment with the curriculum, assessment for and of learning, and leadership are covered. Recently online assessment, the e-portfolio and the use of entrustable professional activities were added to the course content. In the future we would like to accredit more clinical trainers through a process of workplace-based self-evaluation and 360-degree feedback from peers and students. Lastly, we want to expand the training course in the African region.

**Contribution:**

The Train the Clinical Trainer (TCT) course, established in 2014 through RCGP and SAAFP collaboration in South Africa, ensures family physicians have essential educational skills. Aligned with national standards, the course embraces learner-centered principles. Ongoing enhancements include online assessments and future plans involve accrediting more trainers through self-evaluation and expanding across the African region.

## Introduction

The main role of family physicians (FPs), as described in the national position article of the South African Academy of Family Physicians (SAAFP), is to strengthen the district health system.^1^ Family physicians are ideally situated to improve the quality of patient care and promote the safety of patients, through teamwork and capacity building, in district hospitals and primary healthcare. The national Train the Clinical Trainers (TCT) course was introduced in 2014, as a joint venture between the Royal College of General Practitioners (RCGP) and the SAAFP, to address the shortage of FPs and to improve the throughput of the nine South African training programmes. The strategy was to improve the quality of clinical training in the workplace in order to improve the availability and competence of newly qualified FPs in the country.

If you want good clinicians, you need to train them in the same workplace where they will eventually practise independently. Vocational or job training for general practitioners and family medicine registrars is not new. As early as 1985, the Education Committee of the then South African Academy of Family Practice introduced informal vocational training, to address the shortage of doctors in rural areas. This education committee was also responsible for the training of clinical trainers between 1985 and 1990.^2^ However, the recognition of family medicine as a speciality in 2007 led to formal registrar posts and specialist training programmes that required competent clinical trainers.

## Design and development of the course

The vision of the TCT course is to promote and develop postgraduate family medicine training, although the skills are applicable to all workplace-based student training and capacity building of staff. The aim of the 5-day TCT is to equip workplace-based clinical trainers with an essential set of educational skills, which can be further developed through mentoring and support. The annual TCT incrementally creates a critical mass of clinical trainers who are competent and confident to train and assess registrars in the workplace. It also provides an opportunity for the professional development of trainers.

The TCT course is built around the educational principle of learner-centredness. The design enables the course to be flexible, adaptable and context-specific. It was important to adapt the RCGP course to the South African context, where most training takes place on the district health platform. The course was aligned with the National Unit Standards for Family Medicine, as these defined the learning outcomes for registrars and what clinical trainers were attempting to achieve. All assessments, training and course content were designed with the National Unit Standards in mind. The use of assessment tools from the national portfolio of learning for registrars made the training more authentic. The role of the portfolio, to keep track of continuous, reliable and valid workplace-based assessment, is emphasised throughout the course.

The course covers the roles and responsibilities of trainers and learners, the learning environment, alignment with the curriculum, assessment for and of learning, and leadership. Table 1 gives an overview of the course. The importance of constructive alignment between learning outcomes, assessment, teaching methods and content is central to the training. Effective feedback as a learning tool is emphasised and demonstrated throughout the course. This includes feedback on feedback where trainers receive feedback on the way that they give feedback to trainees. On day 4 of the course, participants conduct a brief simulated training session, where they incorporate what they learned and then receive feedback on their training.

**TABLE 1:** An overview of the course.

Session	Topic
**Day 1**	
am	Welcome and orientation
	Exploring different learning styles
pm	Working with adult learners and reflective practice
**Day 2**	
am	Aligning training with the curriculum Use of entrustable professional activities
pm	Establishing a learning environment
**Day 3**	
am	Assessment methods Giving feedback
pm	Use of the e-portfolio (Scorion^®^) and benchmarking
**Day 4**	
am	An introduction to micro-teaching activities
pm	Microteaching (simulation and feedback)
**Day 5**	
am	Small group teaching
	An approach to the learner in difficulty
	Leadership as a clinical trainer
	Closing session

Sessions focus on the learning needs of the learners to support both organisational and professional development. On the final day, participants perform a self-assessment of their expertise as a clinical trainer and create a learning plan for their further development. Participants are provided with all the course materials on a memory stick and if funds allow, they are also provided with the ‘Essential Handbook for GP Training and Education’.^3^

## Implementation

The Education and Training Committee of the SAAFP is responsible for ensuring the quality and content of the TCT course. During training, facilitators (trainers of the course) meet at the end of each day to identify what worked well and what could be done even better. This, together with the daily feedback from the participants (clinical trainers) is then incorporated into the training of the next day and the training plan is updated for future courses. The course facilitators also identify possible new facilitators who can be trained to help with future TCTs. New facilitators are supported by accredited facilitators and perform training under their supervision until they are ready to train on their own.

To ensure equity, each university in South Africa is allowed to send two participants to the TCT course that takes place annually in a central venue. The SAAFP sponsors the costs of the facilitators and running the course, while the universities cover the costs of their participants in terms of travel and accommodation. The small group (18–20 participants) training allows deep learning and is led by 3–4 facilitators. Most participants are initially hesitant to engage in group activities. This is addressed by applying adult learning (andragogical) strategies during theoretical and practical sessions. Adults are internally motivated, goal-orientated and practical; therefore, clinical trainers should focus on facilitating learning rather than being prescriptive.

## Evaluation and revisions

At the end of each course, participants fill in an anonymous evaluation form to give feedback. This feedback includes the relevance of the topics, training methods used and facilitator evaluations. The course coordinator sends individual feedback to each facilitator on their performance and indicates areas of excellence and aspects that should be considered for further development.

Participants are expected to share their personal development plans with their own departments. The intention is that their training sites are then visited by their departments to monitor and support their development as well as assess the learning environment. Unfortunately, this does not always happen.

Two studies have evaluated the TCT. The first study looked at the impact of the TCT by visiting five participants in their workplaces.^4^ The measurement consisted of video recordings of registrar training, a pre-visit self-assessment form, interviews with participants and registrars and an assessment of the learning portfolio of each registrar. The participants identified positive changes after the TCT course as their training became more learner-centred; they structured registrar training better, and they were more confident as clinical trainers. The difficulties with the logistics of site visits were also identified. In the second article, a 360-degree evaluation and self-assessment were performed by trainers after participation in the TCT course. The results showed significant improvement in their clinical training 3 months after the course.^5^

## Conclusion

Currently, the training programmes are adopting an e-portfolio (based on SCORION software) and introducing entrustable professional activities (EPAs) for workplace-based assessment.^6^ The e-portfolio has been included in the TCT to practically demonstrate some of the assessment tools and enable clinical trainers to use the software. By using the tools in a protected environment during the course, trainers gain confidence and a better understanding of concepts, such as benchmarking and competence.

In 2023, the first group of TCT facilitators were accredited by the SAAFP as competent and effective clinical trainers in family medicine.^7^ There is an opportunity for TCT participants to also be accredited as clinical trainers by submitting two reports of satisfactory on-site developmental visits and a 360-degree assessment of their expertise to the Education and Training Committee of the SAAFP. The intention is to incentivise clinical trainers to develop the expertise necessary for accreditation and to receive recognition.

The next step is to provide regular support and updates to the 166 national and 25 international trainees of the TCT course through establishing a special interest group for clinical trainers within the SAAFP. As the training evolves, many previous trainees will need updates on new developments in workplace-based assessment and training. The group will utilise webinars, ad hoc training events and workshops, particularly at the National Family Practitioners Conference, to connect and capacitate clinical trainers.

Through the primary care and family medicine (PRIMAFAMED) network in sub-Saharan Africa,^8^ we would like to expand the training to more African countries. This was done previously through the Family Medicine Leadership, Education and Assessment Programme (FaMLEAP).^9^

In its current format, the TCT course is relevant and sustainable as all training facilities are committed to improving workplace-based training and assessment. The course is ideal for improving both theoretical and practical training practices, as it is very interactive and participants learn from each other. We believe that the training of FPs is in good hands, if we continue to train competent and confident clinical trainers.
